# Prevalence and socio-demographic correlates for serious injury among adolescents participating in the Djibouti 2007 Global School-based Health Survey

**DOI:** 10.1186/1756-0500-4-372

**Published:** 2011-09-27

**Authors:** Adamson S Muula, Seter Siziya, Emmanuel Rudatsikira

**Affiliations:** 1 Department of Community Health, University of Malawi, College of Medicine, Blantyre, Malawi; 2 Department of Community Medicine, University of Zambia, Medical School, Lusaka, Zambia; 3 School of Community Health and Environmental Health, College of Health Sciences, Old Dominion University, USA

## Abstract

**Background:**

Mental health and injury are neglected public health issues especially in low-income nations. The objective of the study was to determine the prevalence and socio-demographic correlates for serious injury in the last 12 months.

**Findings:**

The study used data of the 2007 Djibouti Global School-based Health Survey. Logistic regression analysis was used to establish associations. Of the 1, 777 respondents, 61.1% (63.2% males and 57.8% females) reported having sustained serious injury (SSI). Compared to participants who were not bullied, those who reported being bullied 3-9 days per month were more likely to have sustained serious injury in the last 12 months (AOR = 1.27; 95% CI [1.06, 1.52] for 3-5 days of bullying victimization per month, and AOR = 3.19; 95% CI [2.28, 4.47] for 6-9 days per month. Adolescents who were engaged in physical fighting were 47% (AOR = 1.47, 95% CI [1.40, 1.55] more likely to have sustained serious injury compared to those who were not engaged in the fighting. Meanwhile, adolescents who used substances (cigarettes, other forms of tobacco or drugs) were 30% (AOR = 1.30, 95% CI [1.19, 1.42]) more likely to have sustained serious injury compared to those who did not use substances.

**Conclusions:**

Serious injury is common among adolescents in Djibouti, and we suggest that health workers attending to injured adolescents explore the patients' psycho-social environment. Further, we suggest longitudinal studies where reduction of substance use and bullying may be assessed if they have an impact in reducing serious injury among adolescents.

## Background

Injuries are an important, yet poorly appreciated, cause of morbidity and mortality globally. Both intentional (inter-personal and self inflicted) and unintentional (e.g. the majority arising from road traffic 'accidents') injuries contribute at least 10% of global mortality burden [1]. In a study of serious injury among school going adolescents in six African countries (Kenya, Namibia, Swaziland, Uganda, Zambia, and Zimbabwe), Peltzer [2] found that the mean rate of serious injury was 68.2%, ranging from 38.6% in Swaziland to 71.5% in Zambia. Serious injury among adolescents occurs in diverse circumstances and includes inter-personal, intentional self-harm as well as non-intentional self-harm [3-6]. Serious injury can lead to lifelong consequences for both the victims and their families, especially in countries where specialized trauma centres are not readily available.

Several factors have been identified to be significantly associated with serious injury. In 35 North American and Europe countries, consistently, physical fighting was reported to be significantly associated with elevated risks for medically treated, multiple, and hospitalized injury events among adolescents with between the ages of 11-15 years [7]. Peltzer [2] also reported a significant association between having been in a fight and having sustained a serious injury among adolescents aged 13-15 years in six African countries in eastern, central and southern Africa. In this study an injury was serious if it necessitated missing at least one full day of usual activities (such as school, sports, or a job) or required treatment by a doctor or nurse. In the same study, Peltzer [2] also reported significant associations between being bullied and substance use (smoking, drinking, drug use) on one hand and having sustained a serious injury on the other.

Injuries are an important public health concerns among adolescents. Hammig et al [8] reported in the United States National Longitudinal Study of Adolescents Health that involvement in fights was associated with injuries among the adolescents. In a study of Australian adolescents reported by Chapman et al [9] the commonest among adolescents arising from sports, followed by fights. Alcohol use was most frequently reported to be associated with interpersonal violence injuries. In a 2005 study by Pickett al [10] drunkenness was associated with injuries and fighting among adolescents. Mytton [11] found that the following factors were positively associated with injuries among adolescents: being male, history of previous injury, increasing number of siblings, and neighbourhood disadvantage.

Data on injury and its associated factors among adolescents in low and middle income country are largely lacking. This is much more so in terms on prospective studies [11]. Mytton et al [11] reported that of the 44 eligible studies included in the systematic review, only four studies (from China, Taiwan and Thailand) were reported. Much of the cross sectional studies on injury among prevention in sub-Saharan Africa come from South Africa, Kenya, Nigeria and Ghana.

It is reported in the Djibouti 2007 Global School-based Health Survey Fact Sheet [12] that 60.6% of the students (63.0% of boys and 56.6% of girls) aged 13-15 years were seriously injured one or more times during the previous 12 months to the survey. In that analysis, 48% of the participants aged less than 13 years or aged more than 15 years were excluded from the analysis. Furthermore, factors associated with sustaining serious injury were not analyzed in this study. In addition, Peltzer [2] observed large cross-national variations in the prevalence of serious injury in six African countries and it is thus important that each country documents the epidemiology of serious injury. We, thus, conducted a secondary study using the Djibouti 2007 Global School-based Health Survey to determine the prevalence of serious injury using a larger sample and to determine socio-demographic correlates for serious injury in Djibouti.

## Methods

### Study setting

Data for this study was obtained from Djibouti, a country in the Horn of Africa bordered by Eritrea to the north, Ethiopia in the west and south, and Somalia in the southeast. To the east are the Red Sea and the Gulf of Aden. The country had an estimated population of 740, 528 in 2010 of whom 87% were in urban areas [13].

### Study design

This study was based on a secondary analysis of data from the Djibouti Global School-based Student Health Survey (GSHS) that was conducted in 2007. The 2007 Djibouti GSHS was a school-based survey which included students in classes with the majority ages of 13-15 years (Classe de 5ème, Classe de 4ème, Classe de 3ème). A two-stage cluster sampling design was used to produce data that was representative of students in the country. At the first stage, schools were selected with probability proportional to enrolment size. At the second stage, classes were randomly selected and all students in selected classes were eligible to participate. There was no replacement of students who were not available despite being in the selected schools. The school response rate was 85%, the student response rate was 98%, resulting in an overall response rate of 83%. A total of 1, 777 students participated in the 2007 Djibouti GSHS. Students self-completed the questionnaires and the responses to questions were indicated on a computer scannable answer sheet.

The following question was asked in order to collect data on the type or cause of the most serious injury: During the past 12 months, what was the most serious injury that happened to you? Options were: I was not seriously injured during the past 12 months; I had a broken bone or a dislocated joint; I had a cut, puncture, or stab wound; I had a concussion or other head or neck injury, was knocked out, or could not breathe; I had a gunshot wound; I had a bad burn; I lost all or part of a foot, leg, hand, or arm and; Something else happened to me. In order to assess history of victimization from bullying, the question that was asked was: During the past 30 days, on how many days were you bullied? Possible responses included 0 days or any number of days. In terms of involvement in a physical fight, students were asked the question: During the past 12 months, how many times were you in a physical fight? Options include 0 times, one up to 12 or more times. The question relating to the outcome variable was asked as follows: " During the past 12 months, how many times were you seriously injured? A definition for a serious injury was provided as "An injury is serious when it makes you miss at least one full day of usual activities (such as school, sports, or a job) or requires treatment by a doctor or nurse." Possible responses were 0, 1, 2 or 3, 4 or 5, 6 or 7, 8 or 9, 10 or 11 and 12 or more times.

### Ethical considerations

These data were obtained from the World Health Organization. The study was approved by the Djibouti Ministry of Health. Parents were informed of the study through a letter, and students gave verbal consent to participate in the survey. To preserve individual confidentiality, the questionnaire was anonymously self-reported by the students.

### Data analysis

Cases with missing values were not considered in the analysis. A weighting factor was used in the analysis to account for differences in the probability of selecting a school and classroom, and to account for differences in non response rates for various strata. The weight used in the analysis was calculated using the formula: W = W1*W2*f1*f2*f3*f4, where W1 is the inverse of the probability of selecting a school, W2 is the inverse of the probability of selecting a classroom within a selected school, fl is a school-level non-response adjustment factor calculated by school size category (small, medium, large), f2 is a class-level non-response adjustment factor calculated for each school, f3 is a student-level non-response adjustment factor calculated by class, and f4 is a post-stratification adjustment factor calculated by grade.

Data were first analyzed using SUDAAN software (Research Triangle Park, North Carolina, United States), and reviewed using SPSS version 11.5. Unweighted frequencies and their weighted percentages are reported. For the main outcome (most serious injury), we recoded the responses in two categories: 0 for "I was not seriously injured during the past 12 months", and 1 for other responses. We conducted bivariate and multivariate logistic regression analyses in order to estimate associations between independent factors and the outcome variable. The hypotheses were "There were no significant associations between socio-demographic factors and serious injury". We report prevalence of serious injury as well as the unadjusted odds ratios (OR) and adjusted odds ratios (AOR) together with their 95% confidence intervals (CI).

## Results

### Characteristics of the study sample

A total of 1, 777 students participated in the study of which 60.2% were males. Most (43.2%) of the participants were aged 16 years or older. Overall, 57.6% of the participants (63.9% of males and 48.1% of females) reported having been involved in physical fighting in past 12 months, while 42.1% were bullied in the last 30 days preceding the survey. Substance use (cigarette smoking, other forms of tobacco or drugs) was reported by 15.1% of males and 8.6% of females. Overall, 61.1% of the participants (63.2% of males and 57.8% of females) reported having sustained serious injury. These results are shown in Table 1.

**Table 1:** Socio-demographic and injury characteristics for the students who participated in the 2007 Djibouti Global School-based Health Survey

Factor	Total **n**^**1 **^**(%)**^**2**^	Males **n**^**1 **^**(%)**^**2**^	Females **n**^**1 **^**(%)**^**2**^
Age (years)			
< 14	180 (10.8)	94 (9.7)	86 (12.4)
14	315 (18.2)	181 (17.9)	134 (18.8)
15	498 (27.8)	295 (28.8)	202 (26.2)
16+	718 (43.2)	402 (43.6)	316 (42.6)
			
Gender			
Male	1013 (60.2)	-	-
Female	761 (39.8)	-	-
			
Bullied (number of days during past 30 days)			
0	790 (57.9)	442 (56.9)	348 (59.7)
1-2	271 (20.1)	148 (19.7)	121 (20.5)
3-5	125 (9.1)	76 (9.7)	48 (7.9)
6-9	57 (4.2)	30 (3.9)	27 (4.7)
10 or more	116 (8.8)	73 (9.9)	43 (7.1)
			
Involved in physical fighting in past 12 months			
No	760 (42.4)	367 (36.1)	391 (51.9)
Yes	1009 (57.6)	642 (63.9)	366 (48.1)
			
Substance use (cigarette smoking, other forms of tobacco or drugs)			
No	1444 (87.5)	783 (84.9)	658 (91.4)
Yes	198 (12.5)	135 (15.1)	63 (8.6)
			
Seriously injured*			
No	536 (38.9)	292 (36.8)	244 (42.2)
Yes	833 (61.1)	495 (63.2)	336 (57.8)

n^1 ^unweighted frequency; (%)^2 ^weighted percent.

Frequencies may not add up due to missing information

* serious injury was defined as an injury that made a student to miss at least one full day of usual activities (such as school, sports, or job) or required treatment by a doctor or nurse.

### Common serious injury among adolescents in Djibouti

The most common serious injury among males were cut, puncture, or stab wound (19.5%) and gunshot (17.4%). Broken bone or dislocated joint (21.1%), and cut, puncture, or stab wound (18.8%) were the most common serious injury reported by females (Table 2).

**Table 2:** Frequency of the most serious injury among adolescents who participated in the 2007 Djibouti Global School-based Health Survey

Type of serious injury	Total **n**^**1 **^**(%)**^**2**^	Males **n**^**1 **^**(%)**^**2**^	Females **n**^**1 **^**(%)**^**2**^
Gunshot wound	81 (16.2)	50 (17.4)	31 (14.5)
Concussion or other head or neck injury	61 (12.0)	41 (14.1)	20 (8.8)
Broken bone or dislocated joint	90 (17.8)	45 (15.7)	45 (21.1)
Cut, puncture, or stab wound	104 (19.4)	62 (19.5)	41 (18.8)
Loss of all or part of a food, leg, hand, or arm	24 (4.4)	9 (2.9)	15 (6.7)
Bad burn	38 (6.9)	21 (6.6)	17 (7.4)
Other types of serious injuries	116 (23.4)	67 (23.8)	49 (22.8)

n^1 ^unweighted frequency; (%)^2 ^weighted percent.

Frequencies may not add up due to missing information

Serious injury was defined as an injury that made a student to miss at least one full day of usual activities (such as school, sports, or job) or required treatment by a doctor or nurse.

### Correlates of serious injuries among adolescents in Djibouti

Table 3 shows factors associated with serious injury. All the factors considered in the study were significantly associated with serious injury in bivariate analyses. However, gender was no longer significantly associated with serious injury in a multivariate analysis. Compared to participants who were of age less than 14 years, participants who were aged 14 years (AOR = 0.89, 95%CI [0.81, 0.98]) and those who were of age 16 years or older (AOR = 0.82, 95%CI [0.76, 0.89]) were less likely to have sustained serious injury. Compared to participants who were not bullied, those who experienced 3-9 days of bullying victimization per month were more likely to have sustained serious injuries in the last 12 months (AOR = 1.27; 95% CI [1.06, 1.52] for 3-5 days per month, and AOR = 3.19; 95% CI [2.28, 4.47] for 6-9 days per month). Adolescents who were engaged in physical fighting were 47% (AOR = 1.47; 95% CI [1.40, 1.55] more likely to have sustained serious injury compared to those who were not engaged in the fighting. Meanwhile, adolescents who used substances were 30% (AOR = 1.30, 95% CI [1.19, 1.42]) more likely to have sustained serious injury compare to those who did not use substances.

**Table 3:** Factors associated with most serious injury among adolescents who participated in the 2007 Djibouti Global School-based Health Survey

Factor	Group specific serious injury **n**^**1 **^**(%)**^**2 **^	OR^3 ^(95% CI^4^)	AOR^5 ^(95% CI)
Age (years)			
< 14	180 (66.6)	1	1
14	315 (58.6)	0.87 (0.81, 0.94)	0.89 (0.81, 0.98)
15	498 (61.3)	0.98 (0.91, 1.05)	0.94 (0.86, 1.02)
16+	718 (60.8)	0.96 (0.90, 1.12)	0.82 (0.76, 0.89)
			
Gender			
Male	1013 (63.2)	1	1
Female	761 (57.8)	0.89 (0.86, 0.93)	0.96 (0.92, 1.01)
			
Bullied (number of days during past 30 days)			
0	790 (44.8)	1	1
1-2	271 (73.6)	0.83 (0.73, 0.94)	0.99 (0.86, 1.13)
3-5	125 (81.4)	1.31 (1.11, 1.55)	1.27 (1.06, 1.52)
6-9	57 (93.1)	4.00 (2.88, 5.56)	3.19 (2.28, 4.47)
10 or more	116 (76.3)	0.96 (0.81, 1.13)	0.84 (0.71, 1.00)*
			
Involved in physical fighting in past 12 months			
No	760 (47.5)	1	1
Yes	1009 (70.1)	1.61 (1.55, 1.67)	1.47 (1.40, 1.55)
			
Substance use (cigarette smoking, other forms of tobacco or drugs)			
No	1444 (57.0)	1	1
Yes	198 (76.6)	1.57 (1.47, 1.68)	1.30 (1.19, 1.42)

n^1 ^(%)^2^; OR^3 ^unadjusted odds ratio; CI^4 ^confidence interval; AOR^5 ^Adjusted odds ratio

* p = 0.054

* serious injury was defined as an injury that made a student to miss at least one full day of usual activities (such as school, sports, or job) or required treatment by a doctor or nurse.

## Discussion

In a study of school-going adolescents in Djibouti, East Africa, in which self-reports of serious injury were made, we found that 61.1% participants (63.2% of males and 57.8% of females) reported to have sustained serious injury in the past 12 months. The prevalence rate in this study is similar to 68.2% reported by Peltzer [2] from a study of injury among in-school adolescents in six African countries (Kenya, Namibia, Swaziland, Uganda, Zambia, and Zimbabwe), despite differences in the age groups that were studied. Peltzer [2] considered only participants who were in the age group 13-15 years, while in our case we included participants who were below the age of 13 years and those who were older than 15 years. The prevalence of serious injury in the current study is generally higher than those found in 35 North American and European countries of 33-62% among males and 19-39% among females [10]. Differences in the prevalence rates between the studies may partly be explained by the difference in the definition of serious injury. In our study and in Peltzer's study [2] serious injury was defined as an injury that would make a student miss at least one full day of usual activities (such as school, sports, or a job) or require treatment by a doctor or nurse. Meanwhile, serious injury in the study by Pickett et al [11] was defined as an injury that would require medical treatment.

In a multinational study, Peltzer [2] reported a significant association between having been in a fight and having sustained a serious injury among adolescents aged 13-15 years in six African countries in eastern, central and southern Africa. Associations between victimization from bullying and substance use (smoking, drinking, drug use) on one hand and sustaining serious injury on the other have been reported by Pickett et al [6,10] and Peltzer [2]. We found that adolescents who were substance users (cigarette smoking, other forms of tobacco or drugs), or had engaged in physical fighting or had been victimized by bullies were more likely to report serious injury compared to those with no such experiences.

As this study was based on cross sectional data, we do not assign causation to any of the variables associated with the outcome (self-reported experience of a serious injury). The association between substance use and serious injury experience could have arisen due to multiple pathways. Substance use could be an indication of lack of parental supervision. Adolescents who have limited parental supervision could be exposed to hazardous socio-physical environments where serious injury is likely to occur. We also did not have information as to whether substance use was initiated before injury or occurred following the injury.

Adolescents who reported having engaged in physical fight reported to have experienced serious injury. While some of the adolescents may have sustained serious injury from the fight, others may have resorted to fighting following victimization from bullying as a matter of self-defence or altered conflict resolution behaviour [14]. Whichever the case may be, we suggest that health professionals providing care to injured adolescent explore the possibility of multiple problems (e.g. substance use, bully victimization, and parental supervision).

### Limitations of the present study

The present study has several limitations. Data were collected from a cross sectional study and so any relationship between the outcome and explanatory variables may not be causal. Secondly, data were self-reported and there was no validation of reported serious injury with parental interviews, health insurance or medical records. Thirdly, it is possible there were unmeasured confounders which we could not control for in multivariate analysis. For example, some studies have reported that family circumstance, being a bully, being heavy in stature or shorter, maternal and adolescents' psychosocial problems, developmental challenges were associated with injuries [15-18]. These, and many other factors were not associated in the present study, and therefore, could present residual confounding. In addition, the findings may be applicable and generalized to school-going children in the sampled age groups but may not thus be generalized to all children in the age group surveyed who did not attend school. We conducted a complete case analysis. If those adolescents with missing data were systematically different from those with complete data In addition data arose from the nature of the questions asked in the questionnaire. For instance the question on serious injury presented the options of serious injury experience both by mechanism (gunshot wound) as well as organ or site of the body affected (neck, head) as if these are always mutually exclusive. An individual who may have sustained a gunshot might have sustained this to the head, but the option to present both pieces of information was not possible. Finally, we did not assess the economic and social cost of the reported injury. The contribution of the burden of injuries to reduced development and hospital care in this community is therefore not known from the data available in this study.

## Conclusions

In a study of school-going adolescents we found about 6 in 10 of the adolescents reported to have sustained serious injury in the past 12 months. Report of serious injury was associated with history of having engaged in physical fighting, substance use or having been bully victimized. We suggest that health workers attending to injured adolescents explore the patients' psycho-social environment. Furthermore, we suggest longitudinal studies where reduction of substance use and bullying may be assessed if they have an impact in reducing serious injury among adolescents.

## Competing interests

The authors declare that they have no competing interests.

## Authors' contributions

ASM led the drafting of the manuscript and participated in the interpretation of the findings. ER conducted the data analysis and participated in the interpretation and drafting of the manuscript, SS reviewed the data analysis, participated in the interpretation of the findings and drafting of the manuscript. All authors read and approved the final manuscript.
